# Persistence of Hyperinvasive Meningococcal Strain Types during Global Spread as Recorded in the PubMLST Database

**DOI:** 10.1371/journal.pone.0045349

**Published:** 2012-09-28

**Authors:** Eleanor R. Watkins, Martin C. J. Maiden

**Affiliations:** Department of Zoology, University of Oxford, Oxford, United Kingdom; Melbourne School of Population Health, Australia

## Abstract

*Neisseria meningitidis* is a major cause of septicaemia and meningitis worldwide. Most disease in Europe, the Americas and Australasia is caused by meningococci expressing serogroup B capsules, but no vaccine against this polysaccharide exists. Potential candidates for ‘serogroup B substitute’ vaccines are outer membrane protein antigens including the typing antigens PorA and FetA. The web-accessible PubMLST database (www.pubmlst.org) was used to investigate the temporal and geographical patterns of associations among PorA and FetA protein variants and lineages defined by combinations of housekeeping genes, known as clonal complexes. The sample contained 3460 isolates with genotypic information from 57 countries over a 74 year period. Although shifting associations among antigen variants and clonal complexes were evident, a subset of strain types associated with several serogroups persisted for decades and proliferated globally. Genetic stability among outer membrane proteins of serogroup A meningococci has been described previously, but here long-lived genetic associations were also observed among meningococci belonging to serogroups B and C. The patterns of variation were consistent with behaviour predicted by models that invoke inter-strain competition mediated by immune selection. There was also substantial geographic and temporal heterogeneity in antigenic repertoires, providing both opportunities and challenges for the design of broad coverage protein-based meningococcal vaccines.

## Introduction

As a leading cause of bacterial meningitis and sepsis worldwide, *Neisseria meningitidis* is responsible for appreciable levels of morbidity and mortality, with 500,000 cases each year [1]. However, despite its overt pathogenicity, *N. meningitidis* evolved as an obligate commensal of the human nasopharynx, with population carriage rates of approximately 10% [2]. Indeed, pathogenesis does not contribute to transmission among hosts, and it is not fully understood as to why these “accidental pathogens” occasionally cause invasive disease. The polysaccharide capsule remains the principal virulence determinant identified to date, and defines the serogroup. Only 5 serogroups (A, B, C, W, Y) are responsible for the majority of invasive disease worldwide, and although protein-polysaccharide conjugate vaccines which target serogroups A, C, W and Y have been developed, such a vaccine against serogroup B is not available, owing to its similarity to the host antigen NCAM [3]. There is urgent need for a substitute for a serogroup B vaccine, as this serogroup is the predominant cause of meningococcal disease in many countries [4]. In addition, there is no vaccine available against serogroup X, which has recently caused disease outbreaks in Africa [5]–[6]. Vaccines based on outer membrane proteins (OMPs), such as outer membrane vesicle (OMV) vaccines, provide an alternative to those which are capsule-based. However, the high rates of horizontal genetic exchange and diversifying selection in meningococcal populations results in antigenic diversity at levels which pose problems for designing universal protein-based vaccines [7]–[8].

Meningococcal genetic diversity, although extensive, is highly structured: the population comprises a number of discrete genetic lineages, recognised as clonal complexes by multilocus sequence typing (MLST) [9]. The majority of disease worldwide is caused by a limited number of these clonal complexes, known as hyperinvasive lineages, each of which is associated with a particular serogroup or small number of serogroups [8]–[11]. Additional discriminatory power for strain typing is provided by antigen-encoding genes, such as the two variable regions of the porin PorA (VR1 and VR2) and the iron-regulated OMP FetA, which has one variable region [12]. In addition to the investigation of disease outbreaks, the study of antigenic typing loci has informed understanding of meningococcal population biology. Marked patterns of population structuring have been observed in the form of non-overlapping combinations of alleles at multiple antigenic loci [13]–[17]. These observations are consistent with theoretical frameworks that posit that pathogen populations are structured by host immune responses into strain types with stable non-overlapping antigenic repertoires [16], [18]. Identical non-overlapping combinations of particular PorA alleles, FetA alleles and clonal complexes have been recorded in isolates collected several years apart, suggesting that strain types which emerge as a result of selective forces of the immune system are stable [17]. Understanding the epidemiological patterns of clonal complexes and OMP alleles is central to planning public health interventions such as OMV vaccination campaigns, as the utility of such vaccines will be determined by the extent of diversity at antigenic loci as well as their stability over time. Indeed, as well as typing loci, PorA and FetA are potential candidates for OMV vaccines, and formulations using PorA have been deployed to target specific serogroup B outbreak strains [12], [19]–[22].

**Figure 1:**
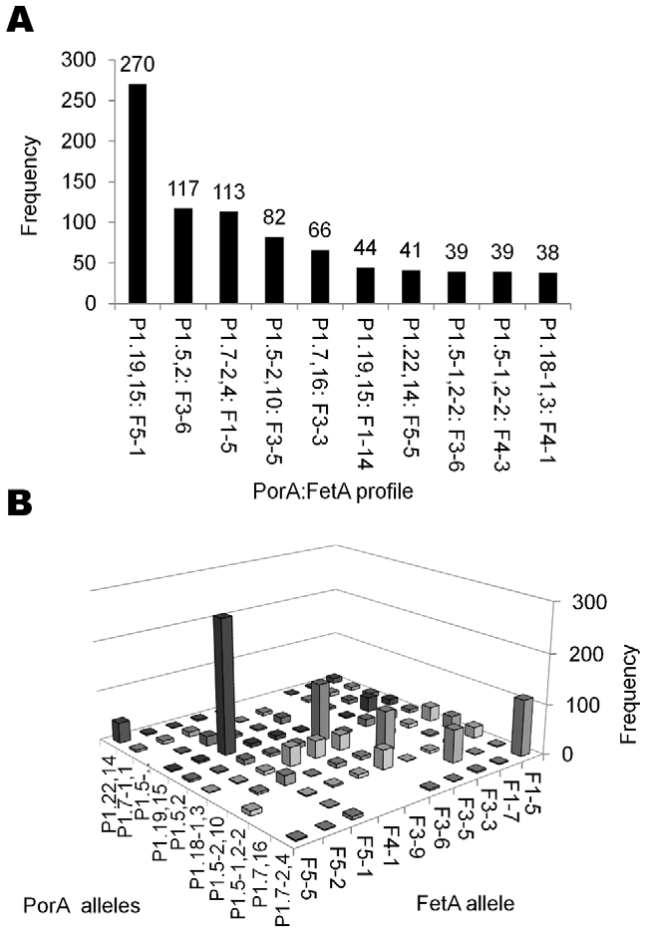
Frequently recorded PorA and FetA variant combinations. The frequencies (A), and asymmetric distribution (B), of the most commonly recorded PorA and FetA allele combinations in the PubMLST database.

The PubMLST database (www.pubmlst.org) is an internet-based repository of bacterial isolate information [23] containing several typing schemes, such as MLST, for a number of species. The database is hosted on publicly-available servers, allowing research communities to access as well as contribute to the genotypic data available [24]–[26]. In addition to MLST data, the *Neisseria* database contains large amounts of genotypic data for the typing antigens PorA and FetA, permitting the investigation of epidemiological patterns among clonal complexes, PorA, and FetA using data collated from a variety of countries over many years. The extensive geographical and temporal sampling frames of the dataset combined with its large sample size provide unique information for understanding global meningococcal population structure and planning vaccine campaigns. Although in its entirety it is not an epidemiologically defined sample, as it comprises isolates that have been voluntarily submitted by the research and public health community, the database does provide a definitive list of described diversity, as submission to it is a prerequisite of sequence type assignment. In addition, the database includes data from various studies with coherent sampling frames which can be extracted and analysed. The PubMLST database therefore enables the investigation of certain defined questions into the population biology and evolution of the meningococcus, e.g. the stability of strain types can be inferred from the time span over which identical combinations of PorA alleles, FetA alleles and clonal complexes have been recorded. Although the formulations for several potential protein-based vaccines contain a number of antigens, including fHBP, NadA and NHBA [27]–[28], these have not been routinely collected in the database: to date, there has been no information on NadA and NHBA allelic variants submitted to the PubMLST database. However, these antigens show similar behaviour to PorA and FetA with respect to their associations with clonal complexes [29]–[30], so conclusions drawn from the study of PorA and FetA are likely to be relevant to understanding their distributions.

Here, the PubMLST database was used to examine the temporal and geographical distributions of associations between clonal complex and the vaccine candidates PorA and FetA, using 3460 carried and invasive isolates from 57 countries representing a 74 year period. The analysis demonstrates that a limited number of PorA:FetA:clonal complex associations from a number of serogroups have persisted for decades and circulated globally. The results raise implications for models of population structure as well as for the design of protein-based vaccines.

**Table 1:** Characteristics of the most frequent strain types in the PubMLST database: many strain types are long-lived and globally widespread.

PorA:FetA:clonal complex	No. isolates	Dominant serogroup(s) (%)^a^	Disease: carriage ratio^b^	Minimum lifespan (years)	Observed time period	Continents found
P1.5,2:F3-6:cc11	117	C (90.6), B (8.5)	1.74	49	1961–2009	Europe, N. America, S. America
P1.5-2,10:F1-5:cc1	19	A (94.7)	8.5	37	1971–2007	Asia, Europe
P1.22,14-6:F1-7:cc41/44	20	B (70), NG (20)	0.05	37	1973–2009	Europe
P1.18-1,3: F4-1:cc22	29	W-135 (70), NG (8)	0.33	36	1975–2010	Europe, N. America
P1.7,16: F3-3:cc32	48	B (93.8)	4.63	35	1976–2010	Europe, Oceania, N. America, S. America
P1.19,15: F1-7:cc41/44	24	B (58.3), NG (33.3)	0.09	31	1980–2010	Europe, Oceania
P1.19,15: F5-1:cc32	263	B (91.6)	1.19	27	1983–2009	Africa, Europe, N. America, S. America
P1.7-2,4: F1-5:cc41/44	105	B (95.2)	32.7	26	1985–2010	Europe, N. America, Oceania, S. America
P1.5-1,2-2: F5-8:cc23	22	Y (90.9)	1.13	26	1985–2010	Africa, Europe, N. America, S. America
P1.5-2,10-1: F4-1:cc23	23	Y (91.3)	0.38	26	1985–2010	Europe, N. America, S. America
P1.18-1,34: F1-9:(-)	22	C (68.2), NG (22.7)	0.16	23	1975–1997	Europe
P1.19,15: F1-14:(-)	40	B (90)	0.67	18	1993–2010	Europe
P1.22-1,14: F4-1:cc35	32	B (84.4), Y (6.2)	0.88	13	1998–2010	Europe, N. America, S. America
P1.18,25-15: F5-5:cc198	22	NG (95.5)	0.1	12	1998–2009	Europe, N. America
P1.5-2,10: F3-5:cc1	80	A (100)	2.81	10	2000–2009	Asia
P1.22,14: F5-5:cc213	40	B (82.5) C (10)	2	8	2003–2010	Asia, Europe, N. America, Oceania
P1.7-2,30: F1-7:cc53	21	NG (90)	0	8	1991–1998	Europe, N. America

a NG refers to non-groupable isolates.

b This ratio describes the proportion of disease and carried isolates found for each strain type as recorded in the PubMLST dataset. It should be noted that, as a result of the nature of the database, these ratios are not necessarily representative and should be interpreted with caution.

**Figure 2:**
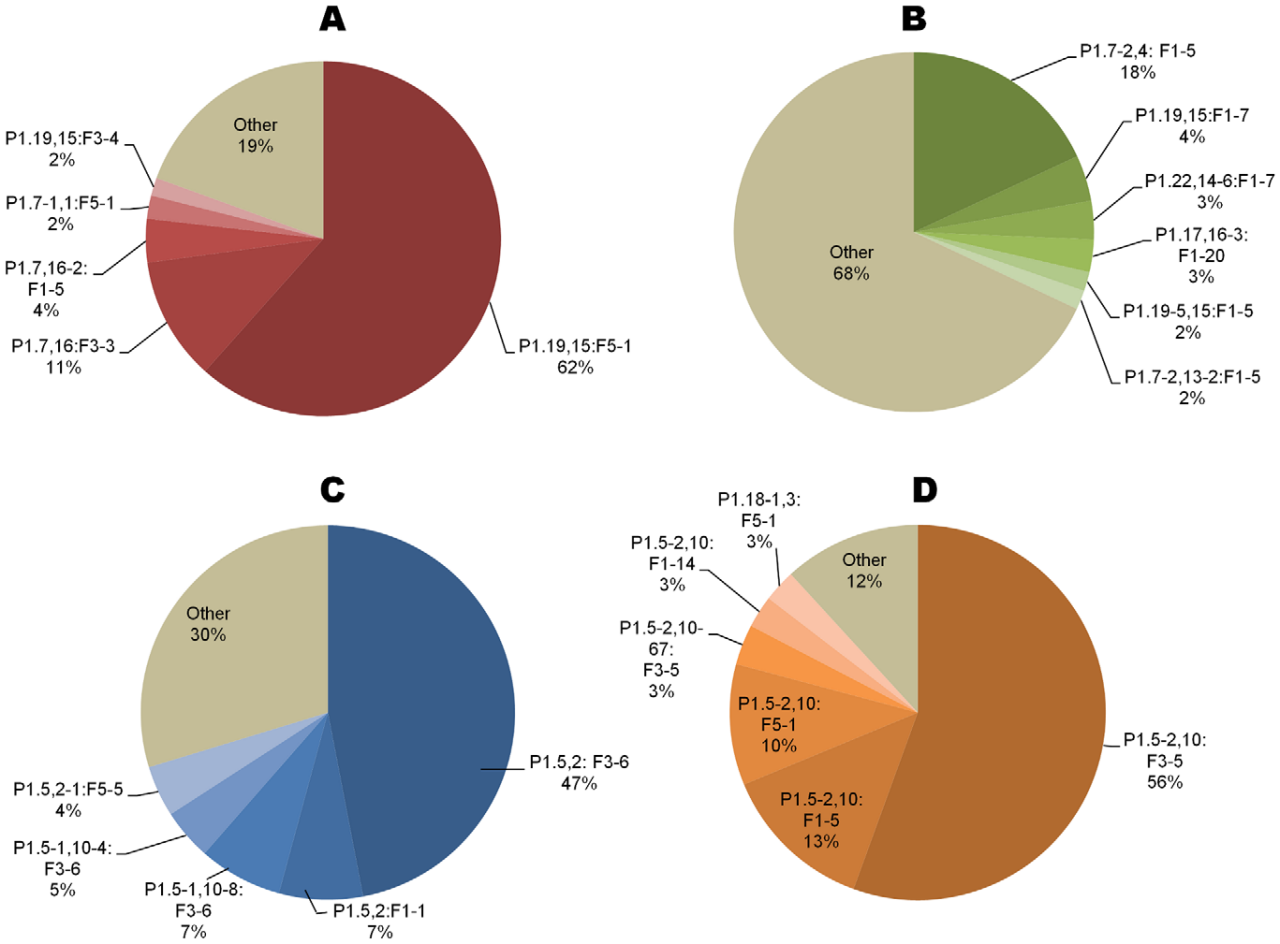
Distribution of PorA:FetA types among four hyperinvasive clonal complexes. A) ST-32 complex, B) ST-41-44 complex, C) ST-11complex and D) ST-1 complex.

**Table 2:** Association of PorA:FetA types with particular clonal complexes.

Antigenic profile	Clonal complex	No. isolates (%)
P1.19,15: F5-1	ST-32	263/270 (97.4)
P1.5,2: F3-6	ST-11	117/117 (100)
P1.7-2,4: F1-5	ST-41/44	105/113 (92.9)
P1.5-2,10: F3-5	ST-1	80/82 (97.6)
P1.7,16: F3-3	ST-32	48/66 (72.7)
P1.19,15: F1-14	Unassigned	40/44 (90.9)
P1.22,14: F5-5	ST-213	40/41 (97.6)
P1.5-1,2-2: F3-6	ST-8	33/39 (84.6)
P1.5-1,2-2: F4-3	ST-549	38/39 (97.4)
P1.18-1,3: F4-1	ST-22	29/38 (76.3)

## Materials and Methods

The PubMLST website [23] is a publicly-available repository of isolate information with an isolate database containing a range of phenotypic and provenance data, linked to sequence-based typing information for various loci. Several sequence typing schemes are embedded in this database, including MLST, antigen and antibiotic sequence typing databases. In each case, records of isolates, allele sequences and schemes (groupings of particular loci) are maintained [31]. The database was searched at 27/10/2010 for all *Neisseria* species isolates containing information on PorA VR1, VR2 and FetA. A total of 3460 isolates had allelic information available for both antigens, dating from 1937 to 2010. More detailed information on the date and location of isolates in the sample is provided as supplementary information (Tables S1 and S2). Internal online database tools were used for data analysis searches, e.g. the “Publication” filter was employed to acquire information from specific published studies.

The nomenclature used was the OMP and clonal complex components recommended by Jolley et al. [32], who suggest: serogroup:PorA type:FetA type:sequence type(clonal complex), thus: B:P1.19,15:F5-1:ST-33(cc32). P1 is a convention maintained from the serosubtyping scheme. Here we use: PorA type:FetA type:clonal complex, thus: P1.19,15:F5-1:cc32. “Minimum lifespan” was defined as the total number of years between the first and final year that a PorA:FetA:clonal complex combination was recorded, including the first and final year. Isolates were not necessarily present in every year over which the life span extends; and equally, isolates present in the database for only a short period may well have been circulating for much longer. Although an approximate measure, it does indicate the minimum period of time over which a specific strain type has been definitively recorded, therefore providing information on minimum strain type longevity. It is highly unlikely that multiple meningococci with identical clonal complexes, PorA alleles and FetA alleles arose independently owing to the large amount of diversity at the antigenic loci, with more than 135 unique peptide sequences for PorA VR1, 375 for PorA VR2 and 186 for FetA [32].

**Figure 3:**
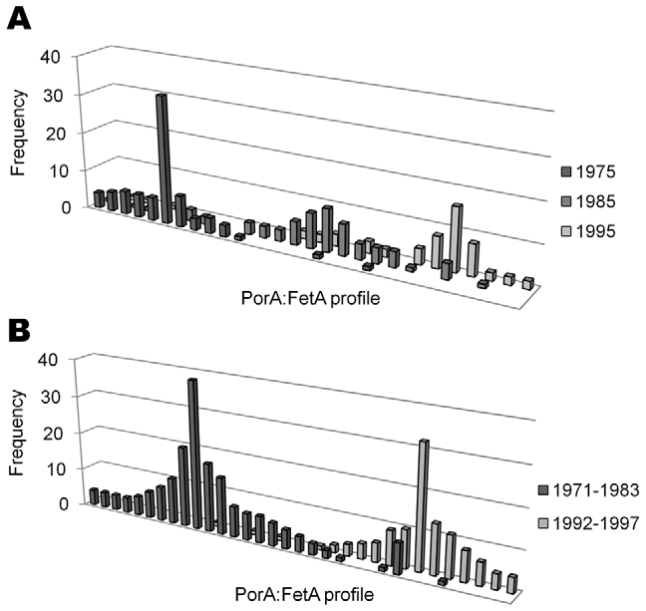
Temporal succession of PorA:FetA profiles in 2 countries. A) PorA:FetA combinations for 207 disease isolates recorded in the United Kingdom in 1975, 1985 and 1995. (Data taken from Russell et al., 2008). B) PorA:FetA combinations for 314 carried isolates in the Czech Republic collected from 1971–1983, and 1992–1997. (Data taken from Buckee et al., 2008).

**Figure 4:**
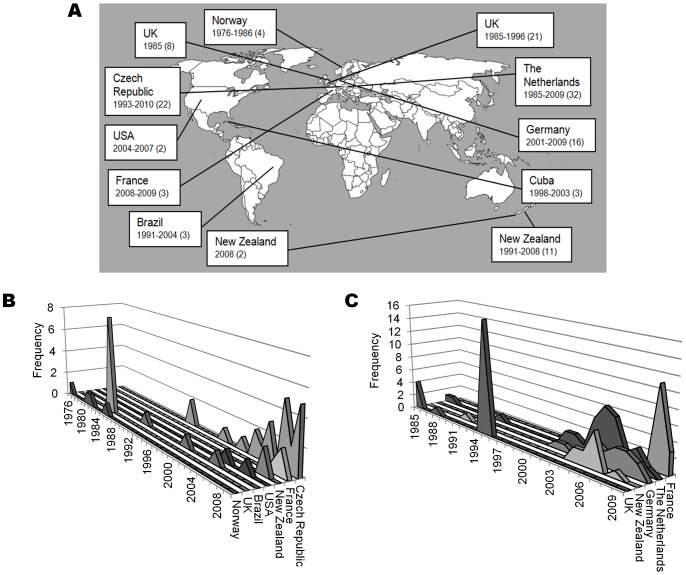
Tracking the global distributions of two hyperinvasive strain types over time. The location, date and frequency of P1.7,16:F3-3:cc32 isolates (A: left side)(B) and P1.7-2,4:F1-5:cc41/44 isolates (A:right side)(C), as reported in the PubMLST database from various countries.

**Table 3:** Hypothetical vaccine coverage conferred by OMV vaccines against isolates in the PubMLST sample.

Trial	Vaccine	Antigens	No. Isolates in dataset (%)	Minimum utility of vaccine (years)*	Ref
Norway	MenBVac	P1.7,16:F3-3	66 (1.9)	35	[19]
New Zealand	MeNZB	P1.7-2,4	147 (4.3)	26	[20]
Cuba	VA-MENGOC-BC	P1.19,15:F5-1	270 (7.8)	36	[21]

* Minimum utility was defined as the total period of time across which a given antigenic combination had been recorded in the PubMLST sample.

## Results

### Diversity of PorA and FetA Associations

A total of 88 PorA VR1 alleles, 223 VR2 alleles and 212 FetA alleles were present in 1129 PorA:FetA combinations in the dataset, of which 757 (67.1%) were present once. There was an asymmetric distribution among PorA:FetA combinations, with a subset recorded at higher frequencies: 13 PorA:FetA types had frequencies exceeding 30 isolates, such as P1.19,15:F5-1, represented by 270 isolates (Figure 1a). Furthermore, several PorA profiles were recorded predominantly with unique FetA profiles not shared by other PorA variants (Figure 1b). For example, 66 of 76 (86.8%) P1.7,16 isolates were associated with F3-3.

### Diversity of PorA, FetA and Clonal Complex Associations

A total of 1420 PorA:FetA:clonal complex combinations were present in the dataset, of which 1030 (72.5%) appeared once. Several strain types were reported at high frequencies, with 10 PorA:FetA:clonal complex combinations represented by over 30 isolates, such as P1.19,15:F5-1:cc32 (263 isolates) and P1.5,2:F3-6:cc11 (117 isolates) (Table 1). Among the more frequent strain types, each PorA:FetA type was recorded predominantly with one clonal complex (Table 2), with a mean of 1.33 clonal complexes found with each PorA:FetA variant combination. For example, all of 117 isolates exhibiting P1.5,2:F3-6 were associated with the ST-11 complex. Similarly, each clonal complex was dominated by a unique antigenic type not shared by other complexes (Figure 2). The ST-41/44 complex, however, was particularly diverse as reflected by its association with 249 variant combinations. A total of 265 of 375 (70.7%) PorA:FetA:clonal complex associations recorded twice or more were associated with one specific serogroup, such as P1.5-2,10:F3-5:cc1, for which all 80 isolates were serogroup A (Table 1). Others were recorded with multiple serogroups, with 14 strain types recorded with three or more different serogroups. For example, P1.5,2:F1-1:cc11 (17 isolates), was recorded with serogroup W-135 (11 isolates), serogroup C (4) and serogroup B (2).

### Prevalence of Strain Types Over Time

Minimum lifespans of strain types, calculated from isolate information in the PubMLST database, ranged from 74 years (of strain type P1.5-2,10:F1-5:cc4) to one year, which was the case for numerous types. A total of 1098 of 1420 (77.3%) PorA:FetA:clonal complex associations had observed lifespans of one year or less; however, these potentially short-lived variants comprised only 1280 out of 3460 isolates (37.0%). The dataset also contained a small number of variants with evidence for intermediate longevity (72 associations persisted for a minimum of 5–9 years), and a larger number of long-lived associations (132) with minimum lifespans in excess of 10 years. Of these, 44 variants had minimum lifespans of 20–39 years and 10 lasted for over 40 years; evidence for longevity was present for only a small minority of strain types.

### Spatio-Temporal Patterns within Countries

Data from coherent population samples from the United Kingdon and Czech Republic stored within the PubMLST database demonstrated that within each country antigenic types showed temporal variation with particular PorA:FetA combinations rising and falling in dominance (Figure 3) [14], [33]. Strain types represented by 2 or more isolates were found in these countries for mean durations of 8.6 and 6.4 years respectively. Each PorA profile was mainly recorded with a particular FetA allele in a given year, which changed over time. For example, F1-5 allele in the United Kingdom was associated with P1.5-1,2-2 in 1975 (8 isolates), P1.7,16-2 in 1985 (11) and P1.7-2,4 in 1995 (16). Fluctuating associations were also seen between clonal complex and PorA:FetA types, e.g. the ST-11 complex in the United Kingdom was associated with P1.5,2:F1-1 in 1975 (6 isolates); P1.5,2-1:F5-5 in 1985 (6); and P1.5,2:F3-6 (8) and P1.5-1,10-4:F3-6 (8) in 1995.

### Global Distribution of Strain Types

Several strain types had representatives in the database from a range of countries across the globe (Table 1), such as P1.7,16:F3-3:cc32 and P1.7-2,4:F1-5:cc41/44, each recorded in 11 countries across Europe, the Americas and Oceania (Figure 4). Many of these strain types had minimum lifespans greater than 10 years and belonged to known hyperinvasive clonal complexes [34]: strain types P1.7,16:F3-3:cc32 and P1.7-2,4:F1-5:cc41/44 had minimum lifespans of 35 and 26 years respectively. The location of isolation of these types changed over time, and they were present in different sites for varying lengths of time (Figure 4).

## Discussion

The application of nucleotide sequence-based typing schemes over the past 20 years has allowed the precise characterisation of meningococcal isolates and has been essential for understanding epidemiological patterns in this diverse pathogen. Targeting the lineages defined by combinations of housekeeping alleles through MLST, together with the typing antigens PorA and FetA, provides a highly discriminatory typing scheme which is reproducible, comprehensive and portable [32]. Moreover, PorA and FetA are candidates for OMV vaccines that, in the absence of capsule-based vaccines, could be deployed against serogroup B meningococci [12], [19]–[22]. The PubMLST site contains information on clonal complex, PorA and FetA, collated from a number of sources worldwide over several years for a large number of isolates [23]. Submission to the database is voluntary, thus the availability of data is dependent upon the motivation of the academic community to undertake such work, as well as the technological and financial capacity to do so, which constrains the amount of data submitted in more resource-poor settings. Although it is not an epidemiologically representative dataset, it still provides information about the location of specific allelic associations at given time points. The sum of genotypic information across the whole PubMLST database demonstrate the stability of strain types and traces their global movements, and are complementary to the published datasets the database contains, with detailed information from individual countries. Here, the temporal and geographical distribution of PorA, FetA and clonal complex associations were investigated in the PubMLST database, using 3460 isolates over 74 years from 57 countries.

The meningococcus is highly genetically diverse, with 1030 unique strain types defined by PorA, FetA and clonal complex present in the database (corresponding to 72.5% of recorded isolates). These assorted combinations of different alleles are consistent with the large amounts of recombination observed in meningococcal populations. However, this diversity was highly structured, as populations typically comprise a limited number of strain types with non-overlapping combinations of outer membrane antigens (Table 1; Table 2; Figure 1). Non-overlapping repertoires of several meningococcal OMP variants have been observed previously, including PorA VR1, VR2, PorB, FetA and the Opa proteins [13]–[14], [16]–[17], [35]. These patterns could be a result of clonal descent or adjacency on the meningococcal chromosome, but owing to the large distance between these loci, the diversity of associations observed, and the extensive horizontal genetic exchange among meningococci, these explanations are unlikely [7]–[8], [36]. Alternative explanations for this structure invoke immune selection [16], [18].

The PubMLST records demonstrated that a number of strain types persisted for several decades (Table 1): a total of 54 PorA:FetA:clonal complex associations were found in meningococci isolated over periods in excess of 20 years. Although longevity of strains has been shown elsewhere [14], [17], [37]–[38], to our knowledge this is the first time that extensive minimum lifespans of up to 74 years have been documented. Furthermore, long-term antigenic stability among meningococci has primarily been shown in isolates belonging to serogroup A [39]. Many of the long-lived associations shown here involve meningococci belonging to serogroups B and C. This is especially intriguing for serogroup B meningococci, which are regarded as highly antigenically diverse [40]. Several of these long-lived strain types were associated with particular hyperinvasive clonal complexes and have spread to a number of countries worldwide (Table 1). Their distribution appears to have shifted between continents over the past few decades, perhaps due to changing host immunity (Figure 4).

The intercontinental spread of several clones of the ST-32 (ET-5) complex was first described in the 1980s [37]; this complex comprises several strain types (Figure 2), and the global dissemination of the serogroup B strain type P1.7,16:F3-3:cc32 is highlighted here. The PubMLST database included records of isolates from widespread locations from 1976 to 2010 (Figure 4a, 4b). Consistent with epidemics in Norway from 1974 onwards, which prompted the development of the MenBVac OMV vaccine, the database showed records in Norway from 1976 to 1986 [19]. A closely related strain type was subsequently reported in the United Kingdom with high frequencies in 1985, in line with a well-characterised outbreak in Gloucestershire at that time [41]; more recently, isolates have been recorded in the United States and France, corresponding to outbreaks in Oregon and Normandy [42]–[43]. Similarly, the ST-41/44 complex hyperinvasive strain type, B:P1.7-2,4:F1-5:cc41/44, was present in 11 countries worldwide over 26 years (Figure 4a, 4c). The strain type was recorded at high frequencies in the United Kingdom in 1995, and more recently in the Netherlands, Germany, New Zealand and elsewhere [20], [44]. A subsequent decline in New Zealand accompanied the immunisation programme of the MenZB vaccine in 2004, which has been suggested as a possible means to control outbreaks in Germany [20], [44].

In addition to global cycling of pandemic meningococci among continents, the PubMLST data suggested a concurrent turnover of strain types within countries. The repertoire of antigenic variants present in each country changed over time with different profiles rising and falling in dominance (Figure 3). There are two factors which could help explain this regional instability. First, these patterns could partly be accounted for by antigenic shifts of endemic strains, an idea supported by molecular data from the United States [45], and fluctuating associations between PorA and FetA observed here and previously [17]. The second explanation is the movement of pandemic hyperinvasive lineages among countries, with host populations colonised by meningococci expressing previously unseen antigenic repertoires. In both cases, the initial increase in frequency of the novel strain type would be a consequence of the lack of population immunity to the strain type, followed by a decline a few years later as immunity to it increased in the host population. For example, the pandemic strain type P1.5,2:F3-6:cc11 expressed PorA and FetA alleles previously unobserved in the Czech Republic when it was introduced following the Velvet Revolution in 1993. Consequently, immediately following its appearance, this strain type caused elevated levels of carriage that accompanied a disease outbreak [33].

Although clonal models of descent have been invoked to explain serogroup A population structure [46]–[47], a number of competing theoretical frameworks have been invoked to explain the population structure of other serogroups, which contain conflicting signals of genetic exchange and clonal descent. The epidemic model [48] posits that clonal expansion can occur within recombining populations if the founding genotypes have a selective advantage. However, the long-term persistence of clonal complexes and antigen combinations observed is inconsistent with this model (Table 1) [13]–[14], [17], [33], [37], [49]–[50]. Alternatively, the immune selection hypothesis [16], [18] predicts the emergence of non-overlapping antigenic repertoires among populations, as strains which share antigenic variants are disadvantaged. These predictions are consistent with the non-overlapping patterns exhibited by PorA and FetA variants shown in the PubMLST dataset and elsewhere [13]–[17]. However, this model does not explain the stable and shifting associations among antigenic types and clonal complexes observed here. A mathematical model which incorporates immune selection and inter-strain competition into the same framework, can account for such patterns [33]. It proposes that competition among lineages is mediated by immune selection, and predicts either stable or shifting non-overlapping associations between antigenic types and sequence types, depending on transmission fitness values simulated. These predictions are also consistent with data from the Czech Republic, which demonstrated both stable and shifting non-overlapping combinations of PorA, FetA and housekeeping alleles over 30 years [17].

Regardless of the mechanisms generating antigenic diversity in meningococcal populations and shaping their structure, this diversity presents several challenges for the design of protein-based vaccines based on variable antigens such as PorA and FetA. First, it is apparently difficult to achieve coverage across many strains [51]: the hypothetical vaccine coverage provided by any of the existing OMV vaccines did not exceed 8% of isolates in the PubMLST dataset (Table 3). Further, a vaccine containing the five most frequent PorA and FetA variants would protect against only 46.9% of meningococci represented in the PubMLST dataset. Second, the temporal dynamics raise problems for coverage, and the propensity to exchange genetic material presents the possibility of antigenic shift among vaccine-target antigens, instances of which have been documented [45]. Third, there is substantial heterogeneity of protein variants among countries at a given point in time (Figure 4), thus hindering the development of a comprehensive vaccine appropriate to all countries. Finally, some evidence suggests that existing protein-based OMV vaccines are poorly immunogenic in infants and may require multiple doses to ensure long-term immunity [52].

Conversely, a number of opportunities for vaccine design are presented by the structuring of meningococcal populations into non-overlapping antigenic variants associated with particular clonal complexes (Figure 2). As a limited number of hyperinvasive strain types circulate at higher frequencies (Table 1) [10], [13], immunisation strategies that target these with vaccines containing appropriate combinations of antigen variants can be devised. Importantly, as several hyperinvasive lineages are associated with more than one serogroup (Table 1) [8]–[11], such vaccines would be effective against these irrespective of the serogroup expressed, potentially circumventing the effects of capsule switching [53]. Secondly, although the diversity of meningococci on a global scale might require a large number of variants for comprehensive coverage, geographic structuring could be exploited in order to design simpler vaccines that are tailor-made to target hyperinvasive lineages within a given locale, as countries typically experience a limited number of circulating hyperinvasive antigenic types at a given time. For example, data from the PubMLST database suggest that a vaccine containing 5 PorA and FetA variants (P1.7-2,4, P1.22,14, P1.21,16, P1.7-2,13-2, P1.22,9; F1-5, F5-5, F1-7, F3-3 and F5-12) would potentially protect against 70.2% of serogroup B strain types in Germany (185 isolates). Antigenic types rise and fall in prevalence over time (Figure 3, Figure 4), but remain present for periods of time sufficient to administer vaccination programmes. This is particularly so in the case of serogroup B outbreaks which can persist for many years [37], such as strain type P1.7-2,4:F1-5:cc41/44, isolated from the database in the Netherlands for at least 25 years. Thirdly, as the minimum lifespans of global hyperinvasive strain types span decades as they move among countries, protein-based vaccines directed against them have the potential to be employed for many years in different places (Table 3). An example of this is the global serogroup B strain type P1.7,16:F-33:cc32, with a minimum lifespan of 35 years, against which the Norwegian vaccine (MenBVac) was developed. The most recent use of this vaccine was in the Normandy region of France, more than twenty years after it was developed [22].

In conclusion, notwithstanding frequent recombination in meningococcal populations, the high level of genetic variation they exhibit is extensively structured. Collections of meningococci are dominated by a relatively small number of discrete combinations of clonal complexes, PorA and FetA variants, which persist for decades and proliferate globally. The long lifespans of these strain types are punctuated by their movements among countries, most likely shaped by dynamics of human herd immunity. This structuring and dynamic behaviour is consistent with mathematical models that invoke selective forces imposed by host immunity to account for pathogen population structure in the face of extensive recombination. It would be interesting to ascertain whether similar temporal and spatial patterns are observed in the outer membrane proteins of other bacterial pathogens, especially those proteins which serve as potential vaccine components. The longevity of the non-overlapping meningococcal strain types, combined with their global circulation, suggest that vaccines that target hyperinvasive lineages based on their antigenic repertoires may hold some promise. The challenge of designing vaccines to tackle meningococci on a global scale perhaps may not be an insurmountable one, even in the face of the diversifying forces of immune selection and recombination.

## Supporting Information

Table S1 **Total number of isolates by country.**
(DOCX)

Table S2 **Total number of isolates by year.** *This lower total is due to a small number of isolates in the database for which no year has been recorded.
(DOCX)
