# Supplementary material for: Persistence of Hyperinvasive Meningococcal Strain Types during Global Spread as Recorded in the PubMLST Database
Source: PLoS One. 2012 Sep 28;7(9):e45349. doi: 10.1371/journal.pone.0045349 (PMC3460945; doi:10.1371/journal.pone.0045349)
Supplement: Table S1 — Total number of isolates by country. (DOCX) [file pone.0045349.s001.docx]

**Table S1. Total number of isolates by country.**

| Country | Frequency |
| --- | --- |
| Czech Republic | 1295 |
| Cuba | 408 |
| UK | 335 |
| The Netherlands | 277 |
| Germany | 265 |
| Russia | 163 |
| France | 128 |
| USA | 117 |
| Brazil | 77 |
| Norway | 72 |
| New Zealand | 49 |
| Poland | 35 |
| Sweden | 28 |
| Spain | 24 |
| Burkina Faso | 20 |
| Mali | 16 |
| Argentina | 12 |
| South Africa | 12 |
| Romania | 12 |
| Gambia | 11 |
| Bangladesh | 10 |
| China | 9 |
| Turkey | 7 |
| India | 6 |
| Senegal | 6 |
| Chile | 6 |
| Unknown | 5 |
| Greece | 5 |
| Denmark | 4 |
| Austria | 4 |
| Australia | 4 |
| Slovak Republic | 3 |
| Italy | 3 |
| Colombia | 3 |
| Morocco | 2 |
| Ghana | 2 |
| Canada | 2 |
| Niger | 2 |
| Iceland | 2 |
| Ireland | 2 |
| Philippines | 1 |
| Congo | 1 |
| Switzerland | 1 |
| Israel | 1 |
| Uganda | 1 |
| Saudi Arabia | 1 |
| Andorra | 1 |
| Costa Rica | 1 |
| Thailand | 1 |
| Korea | 1 |
| Djibouti | 1 |
| Chad | 1 |
| Pakistan | 1 |
| Cyprus | 1 |
| Finland | 1 |
| Sudan | 1 |
| Cameroon | 1 |
| Total | 3460 |
