# Supplementary material for: Persistence of Hyperinvasive Meningococcal Strain Types during Global Spread as Recorded in the PubMLST Database
Source: PLoS One. 2012 Sep 28;7(9):e45349. doi: 10.1371/journal.pone.0045349 (PMC3460945; doi:10.1371/journal.pone.0045349)
Supplement: Table S2 — Total number of isolates by year. *This lower total is due to a small number of isolates in the database for which no year has been recorded. (DOCX) [file pone.0045349.s002.docx]

**Table S2. Total number of isolates by year.**

| Year | Frequency |
| --- | --- |
| 1937 | 1 |
| 1940 | 1 |
| 1941 | 1 |
| 1960 | 4 |
| 1961 | 18 |
| 1962 | 20 |
| 1963 | 29 |
| 1964 | 21 |
| 1966 | 5 |
| 1967 | 3 |
| 1968 | 2 |
| 1969 | 1 |
| 1970 | 2 |
| 1971 | 22 |
| 1972 | 67 |
| 1973 | 43 |
| 1974 | 54 |
| 1975 | 181 |
| 1976 | 7 |
| 1977 | 4 |
| 1978 | 24 |
| 1979 | 20 |
| 1980 | 35 |
| 1981 | 1 |
| 1982 | 8 |
| 1983 | 32 |
| 1984 | 7 |
| 1985 | 134 |
| 1986 | 29 |
| 1987 | 13 |
| 1988 | 33 |
| 1989 | 45 |
| 1990 | 61 |
| 1991 | 57 |
| 1992 | 75 |
| 1993 | 273 |
| 1994 | 13 |
| 1995 | 122 |
| 1996 | 13 |
| 1997 | 34 |
| 1998 | 97 |
| 1999 | 58 |
| 2000 | 35 |
| 2001 | 50 |
| 2002 | 86 |
| 2003 | 51 |
| 2004 | 152 |
| 2005 | 192 |
| 2006 | 226 |
| 2007 | 270 |
| 2008 | 287 |
| 2009 | 303 |
| 2010 | 91 |
| Total | 3413* |
